# A Novel Rotavirus Reverse Genetics Platform Supports Flexible Insertion of Exogenous Genes and Enables Rapid Development of a High-Throughput Neutralization Assay

**DOI:** 10.3390/v15102034

**Published:** 2023-09-30

**Authors:** Jiajie Wei, Scott Radcliffe, Amanda Pirrone, Meiqing Lu, Yuan Li, Jason Cassaday, William Newhard, Gwendolyn J. Heidecker, William A. Rose II, Xi He, Daniel Freed, Michael Citron, Amy Espeseth, Dai Wang

**Affiliations:** 1 Department of Infectious Diseases and Vaccines, Merck & Co., Inc., West Point, PA 19486, USA; amanda.pirrone@merck.com (A.P.); meiqing_lu@merck.com (M.L.); yuan_li@merck.com (Y.L.); jason_cassaday@merck.com (J.C.); william_newhard@merck.com (W.N.); gwendolyn_heidecker@merck.com (G.J.H.); xi_he@merck.com (X.H.); dan_freed@merck.com (D.F.); michael_citron@merck.com (M.C.); espeseth@comcast.net (A.E.); dai_wang@merck.com (D.W.); 2 Department of Quantitative Biosciences, Merck & Co., Inc., West Point, PA 19486, USA; scott.radcliffe@merck.com (S.R.); william.rose@merck.com (W.A.R.II)

**Keywords:** rotavirus, high-throughput screening, microneutralization assay, neutralizing antibody, pre-existing immunity

## Abstract

Despite the success of rotavirus vaccines, rotaviruses remain one of the leading causes of diarrheal diseases, resulting in significant childhood morbidity and mortality, especially in low- and middle-income countries. The reverse genetics system enables the manipulation of the rotavirus genome and opens the possibility of using rotavirus as an expression vector for heterologous proteins, such as vaccine antigens and therapeutic payloads. Here, we demonstrate that three positions in rotavirus genome—the C terminus of NSP1, NSP3 and NSP5—can tolerate the insertion of reporter genes. By using rotavirus expressing GFP, we develop a high-throughput neutralization assay and reveal the pre-existing immunity against rotavirus in humans and other animal species. Our work shows the plasticity of the rotavirus genome and establishes a high-throughput assay for interrogating humoral immune responses, benefiting the design of next-generation rotavirus vaccines and the development of rotavirus-based expression platforms.

## 1. Introduction

Although rotavirus vaccines have substantially reduced rotavirus-related childhood morbidity and mortality worldwide [1], rotaviruses remain one of the most common causes of diarrheal diseases in children, with a higher disease burden in developing countries. Rotaviruses are responsible for 128,500–215,000 deaths annually in children under 5 years old [2,3]. The mechanisms underlying rotavirus vaccine-induced protection are not fully understood, partially due to limitations in current animal models of rotavirus infection and disease. The lower vaccine efficacy observed in low-income countries has been attributed to multiple factors, including higher levels of maternally derived antibodies, different intestinal microbiome resulting from chronic enteropathy, and/or poor nutritional status [4]. To develop the next-generation vaccines with improved safety and efficacy, a better understanding of pre-existing immunity, including neutralizing antibodies in human and animal models and its impact on vaccine efficacy, is needed. 

Rotaviruses are double-stranded, segmented RNA viruses. The 11 genome segments encode 12 viral proteins, including six non-structural proteins (NSP1-NSP6) and six structural proteins (VP1–VP4, VP6, and VP7). Each segment encodes one open reading frame (ORF), except segment 11, with NSP5, which contains an internal ORF for NSP6. Rotavirus particles consist of three concentric proteins shells (VP2, VP6, and VP7) and a spike protein, VP4, which spans the VP6 and VP7 layers and extends out from the particle. Serologically, rotaviruses are grouped into distinct serogroups based on VP6 reactivity. Group A rotaviruses (RVA), further classified into serotypes defined by VP7 (G) and VP4 [P], cause the majority of disease in human. RVA strains are also found in animals, and infection has been shown to be highly species-specific. 

Rotavirus research has been hindered by the lack of a reverse genetics system, which would allow for the generation and engineering of defined viral particles. Since the first publication of a plasmid-only-based reverse genetics system for the simian rotavirus strain SA11 [5], multiple groups have utilized the system to rescue recombinant rotaviruses with different properties. These include the simian RRV strain; human CDC-9 strain; a murine-like RV strain [6]; human Odelia strain [7]; human KU strain [8]; human HN126 strain [9]; bovine RF strain [10]; avian PO-13 strain [11]; chimeric strains with SA11 backbone and VP4, VP7, and/or VP6 genes from human clinical samples [12]; and SA11 carrying NSP2 phosphorylation mutation [13]. Heterologous protein expression from rotavirus was explored by replacing part of the NSP1 ORF with foreign genes [5]. Later, it was shown that genome segment 7 could be re-engineered to encode NSP3 fused to a fluorescent protein [14]. Additionally, domains of SARS-CoV-2 spike protein were also expressed downstream of NSP3 [15], suggesting that rotaviruses may serve as a vector for gene delivery. Recently, the concept of using recombinant rotaviruses expressing norovirus capsid proteins as a dual vaccine was established in an infant mouse model [16]. A solid understanding of pre-existing immunity in human and animal models would further explore the feasibility of using rotavirus to deliver therapeutic proteins or vaccine antigens. 

Here, in addition to the previously published NSP3 site, we have demonstrated two additional genomic locations in NSP1 and NSP5 of the simian rotavirus strain SA11 for expressing heterologous proteins by reverse genetics. We utilized a recombinant rotavirus expressing GFP from NSP1 (rSA11-GFP) to establish a high-throughput rotavirus microneutralization assay. This assay enabled us to determine the presence of pre-existing neutralizing antibodies in human and other animal serum samples. Among the 50 human donor samples [17] that were surveyed, 41 had detectable neutralization titers against SA11, a serotype G3 virus. Additionally, all African green monkeys and rhesus monkeys examined showed pre-existing immunity. In contrast, other animal models, such as rabbit, mouse, guinea pig and cotton rat, either had much lower levels or no detectable neutralizing antibodies. Through the identification of novel approaches for heterologous gene expression, we have demonstrated the plasticity of the rotavirus genome and developed a high-throughput neutralization assay based on a GFP expressing virus. 

## 2. Materials and Methods

### 2.1. Cell Culture

CV1, MA104, and baby hamster kidney cells expressing T7 RNA polymerase (BHK-T7) were maintained in Dulbecco’s Modified Eagle’s Medium (DMEM) with 10% fetal bovine serum (FBS) and 1% penicillin-streptomycin. All cultures were grown at 37 °C in a 5% CO_2_ incubator. 

### 2.2. Plasmid Construction

Sequences of all 16 plasmids used for the generation of the wild-type SA11 strain were obtained from Addgene (https://www.addgene.org/Takeshi_Kobayashi/ (accessed on 29 September 2023)) [5]. pUC19 and pV1Jns served as the backbones of 11 plasmids, each encoding one rotavirus genome segment (pT7/VP1SA11, pT7/VP2SA11, pT7/VP3SA11, pT7/VP4SA11, pT7/VP6SA11, pT7/VP7SA11, pT7/NSP1SA11, pT7/NSP2SA11, pT7/NSP3SA11, pT7/NSP4SA11, and pT7/NSP5SA11) and 5 helper plasmids (pCMV/NSP2, pCMV/NSP5, pCMV/NBVFAST, pCMV/D12L, and pCMV/D1R), respectively. For the expression of GFP, NSP open reading frames were fused with 2A peptide GSGEGRGSLLTCGDVEENPGP and then GFP. After the stop codon, an NSP open reading frame sequence repeat was inserted when indicated. All plasmids were synthesized using Genewiz. 

### 2.3. Recombinant Rotavirus Rescue

Recombinant SA11 (rSA11) strains were generated by reverse genetics, as described previously with modifications [5]. Monolayers of BHK-T7 cells in 6-well plates (1 × 10^6^ cells/well) were used for transfection. 16 plasmids (0.75 µg/plasmid except 0.015 µg pCMV/NSVFAST) in 150 µL of Opti-MEM were added to 150 µL of Opti-MEM containing 12.5 µL of Lipofectamine 2000. Transfection complexes were incubated at room temperature for 20 min and then added to BHK-T7 cells drop-wise. 24 h post transfection, the culture medium was changed into serum-free DMEM. 48 h post transfection, 1.5 × 10^5^ CV1 cells were added to transfected cells, and TPCK-treated trypsin (1 mg/mL stock) was added to culture medium to achieve a final concentration of 1 µg/mL. The transfection reaction was monitored daily and harvested when a complete cytopathic effect (CPE) was observed (typically three days after the addition of CV1 cells). To generate recombinant viruses with heterologous genes, pT7/NSP1SA11, pT7/NSP2SA11, pT7/NSP3SA11, pT7/NSP4SA11, and pT7/NSP5SA11 were replaced with the corresponding plasmid with heterologous gene insertion. rSA11/GFP was then plaque-purified for three rounds on MA104 cells. 

### 2.4. Virus Infection

Recombinant viruses were treated with 10 µg/mL TPCK-treated trypsin at 37 °C for 1 h. Monolayers of CV1 or MA104 cells were washed with serum-free DMEM three times and then infected with trypsin-treated viruses in serum-free DMEM at 37 °C. After 1 h of incubation, inoculums were removed. 

### 2.5. Virus Stock Preparation

MA104 cells were infected with recombinant viruses and cultured in serum-free DMEM containing 1 µg/mL trypsin. Once complete CPE was observed, viruses were harvested by subjecting the infected cells to three freeze–thaw cycles. The virus suspension was then filtered through 0.2 micro filters. Virus concentration and purification were performed using an Amicon Ultra-15 50,000 NMWL centrifugal filter unit. 

### 2.6. Plaque Assay

MA104 cells were infected with recombinant viruses and overlaid with phenol-red-free MEM containing 0.8% agarose and 0.5 µg/mL trypsin. After 4 days of incubation, plaques were visualized by adding 5 mg/mL MTT or picked directly for plaque purification. 

### 2.7. Flow Cytometry and Data Analysis

CV1 cells were counted, infected with recombinant viruses, and cultured in DMEM supplemented with 10% FBS. After overnight incubation, cells were harvested, fixed with 4% paraformaldehyde, and stained with a primary antibody anti-RotaVP6 (UK1, ThermoFisher) and then a secondary antibody Alexa Fluor 647 AffiniPure Goat Anti-Mouse IgG (H + L) (Jackson Immuno Research Labs, West Grove, PA, USA). Staining and washing steps were performed using Perm/Wash buffer (BD Biosciences, San Jose, CA, USA). Flow cytometric data were acquired using a BD LSRII flow cytometer (BD Biosciences, San Jose, CA, USA) and gated on single cells. Data analysis was conducted using FlowJo version 10 software (FlowJo LLC, San Jose, CA, USA). In the flow cytometry-based infectivity assay, the percentage of VP6-positive cell population was numerated by FlowJo. Based on the Poisson distribution, the infectious unit (IU)/mL was calculated as IU/mL = (# of cells at infection) × [MOI/(ml of viral stock used at infection)]. 

### 2.8. Growth Kinetics

MA104 cells were infected with recombinant viruses at a multiplicity of infection (MOI) of 0.01 IU/cell and cultured in serum-free DMEM containing 1 µg/mL trypsin. At 24, 48, and 72 h post-infection, the viruses were harvested by subjecting the infected cells to three freeze–thaw cycles. The virus titer was determined by a flow cytometry-based infectivity assay (Supplementary Figure S1). 

### 2.9. Genetic Stability

Viruses were serially passaged on MA104 cells. Monolayers of MA104 cells were infected with viruses and cultured in serum-free DMEM containing 1 µg/mL trypsin. When complete CPE was observed, the cell culture supernatant was used directly for the next round of infection with 1:1000 final dilution. Viral RNA was extracted from 140 µL of the supernatant using QIAamp viral RNA kit, and 15 µL RNA was used in the SuperScript IV one-step RA-PCR system with forward primer 5′-CAACGGAGGAACTGATTGAAATGAAGAA-3′ and reverse primer 5′-TTGCCAGCTAGGCGCTACT-3′ following manufacturers’ instructions. PCR reactions were analyzed by 1.2% E-gel (ThermoFisher) along with E-Gel 1 Kb Plus Express DNA Ladder. Sanger sequencing reactions were conducted by Genewiz using primers 5′-GCTACTGATCTCCAACTCAGAAGATG-3′ and 5′-TAGTCTGGACGGTCTTGTGA-3′.

### 2.10. ELISA

96-well assay plates were coated with wild-type SA11 (10^5^ PFU/well) in DMEM at 4 °C overnight. The plates were then washed once with 300 µL/well Washing Buffer (PBS + 0.05% Tween 20) and blocked with 200 µL/well Blocking Buffer (Alfa Aesar, Haverhill, MA, USA) at 4 °C overnight. Blocked plates were incubated with a series of 3-fold diluted sera in Blocking Buffer (100 µL/well) at 4 °C overnight. Following sera incubation, the plates were washed three times with 300 µL/well Washing Buffer and incubated with 100 µL/well 1:4000 diluted alkaline phosphatase conjugated Goat anti-Rhesus IgG H&L (used for both African green monkey and human, Southern Tech, Ardmore, PA, USA) in Blocking buffer with 0.1% Tween 20 for 1.5 h at room temperature. After washing the plates three times with Washing Buffer, 100 µL/well Tropix CDP-Star Sapphire II substrate (Applied Biosystem, Waltham, MA, USA) were added. After incubation at room temperature for 10 min, the chemiluminescent signal from each well was read on PHERAstar. The threshold value was 25 times the mean plate background. Interpolated titers were calculated by drawing a line between the last point above the threshold and the first point below the threshold and solving for the fold dilution where that line crosses the threshold. 

### 2.11. rSA11-GFP Based Micro-Neutralization Assay

CV1 cells were seeded into 96-well plate (4 × 10^4^ cells/well) and cultured overnight. Serum samples were heat-inactivated at 56 °C for 30 min and serial diluted in PBS. rSA11-GFP (used at MOI = 1) was activated with 10 µg/mL TPCK-treated trypsin at 37 °C for 1 h and then mixed with serial diluted animal serum samples at 37 °C for 1 h on an orbital shaker. Pre-seeded CV1 cells in the 96-well plates were washed three times with serum-free DMEM, infected with virus/serum mixtures at 37 °C for 1 h, and then cultured in phenol-red-free DMEM supplemented with 10% FBS. For the 384-well neutralization assay, CV1 cells were harvested and washed in serum-free DMEM. 1 × 10^4^ CV1 cells in suspension were added into virus/serum mixtures directly and incubated at 37 °C for 1 h. FBS was then added to the plate to achieve a final concentration of 10%. For both 96 well and 384 well plates, after overnight incubation at 37 °C, plates were read by an Acumen high content screening (HCS) reader (TTP LabTech, Melbourn, UK) at 488 nm to determine numbers of GFP positive cells in each well. The percentages of inhibition were calculated based on control wells to which no animal serum was added. A sample 384-plate map is provided in Supplemental Table S1. The plate had the following characteristics: (1). The first column of the plate was reserved for “no serum” controls. (2). The last column of the plate contained mock infected controls. (3). Serum samples were diluted threefold with an 11-point titration. (4). One serum sample was tested for each animal. (5). Technical duplication was performed for each sample. (6). A positive control, cynomolgus serum sample (enQuire Bioreagents AB155109, Littleton, CO, USA), was included on each plate. This sample had an NT50 value around 100. NT50 was calculated by nonlinear four-parameter curve fitting using Prism 8 (GraphPad, La Jolla, CA, USA) to determine the serum dilution that resulted in a 50% reduction in GFP-positive cells compared to the control. 

### 2.12. Animal and Human Serum Samples

The serum samples used in the assay were obtained from various sources. Human serum samples were reported before [17]. Serum samples from rabbit, mouse, guinea pig and cotton rat were obtained from animals housed in the animal facility in the research laboratories at Merck & Co., Inc., West Point, PA, USA. Simian serum samples were collected from animals at the New Iberia Primate Research Center (NIRC, New Iberia, LA, USA). All studies were conducted in accordance with relevant guidelines using protocols approved by NIRC and the Institutional Animal Care and Use Committee of Merck & Co., Inc., Kenilworth, NJ, USA.

### 2.13. Serum Purification

African green monkey serum was diluted 1:25 in binding buffer and purified using Nab™ Protein G Spin Kit (Thermo Scientific Cat# 89949, Waltham, MA, USA). Diluted samples were incubated in immobilized protein G spin columns at room temperature with end-over-end mixing for 10 min. Spin columns were then centrifuged for 1 min at 5000× *g*, washed three times with binding buffer, and eluted three times with Elution Buffer into Neutralization Buffer via centrifugation. Elution fractions were pooled and concentrated using Amicon Ultra 0.5 mL centrifugal filters.

## 3. Results 

### 3.1. The Utilization of Three Positions in Rotavirus Genome for Heterologous Gene Expression

We aimed to construct rotaviruses expressing heterologous proteins in addition to the full set of rotavirus proteins. A plasmid-only system to rescue simian rotavirus strain SA11 enables the engineering of rotaviruses [5]. Because naturally occurring human rotaviruses that contain rearranged genomes can package up to 1800 additional base pairs in virus particles [18], and rearranged genome segments 7 and 11 with open reading frame (ORF) sequence repeat at the C terminus of NSP3 and NSP5, respectively, are preferentially packaged into rotaviruses [19], we reasoned that these two positions could tolerate foreign gene insertion.

**Figure 1:**
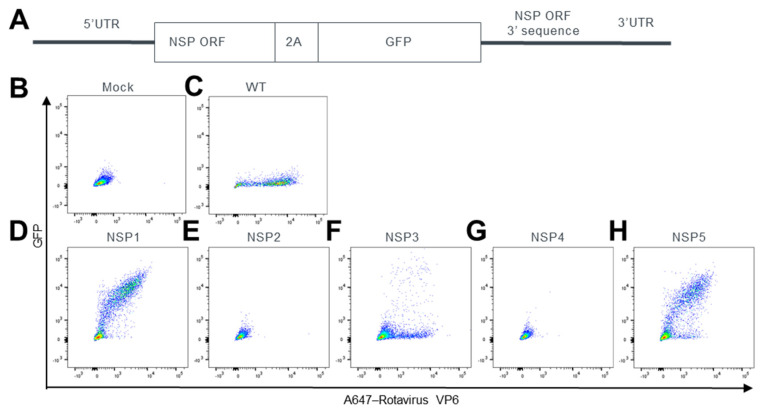
Rescue effort of rSA11 strains expressing GFP from five genome locations. (**A**) Schematic representation of plasmids used for the rescue of rSA11 viruses encoding GFP. (**B**–**H**) Representative flow cytometry analysis of CV-1 cells infected with rSA11 rescue products. GFP sequence was inserted at the indicated positions. Expressions levels of GFP and rotavirus protein VP6 were examined. Pseudocolor plots were shown using color to denote areas of high and low population density.

To systematically explore genomic positions that support heterologous protein expression, we fused a green fluorescent protein (GFP) after a 2A self-cleaving peptide to the C terminus of every nonstructural protein (Figure 1A) except NSP6, given that NSP6 is encoded by an internal ORF inside of NSP5. To keep the potential genome packaging signal at the 3′end of ORF, after the stop codon, a 450 bp fragment of the NSP ORF 3′ sequence was repeated upstream of the 3′UTR. 

We tested each modified genome segment by replacing the corresponding wild-type (WT) SA11 genome segment in the reverse genetics system [5]. We transfected cells with plasmids, used supernatant to infect the CV-1 cells, and then determined the expression levels of GFP and rotavirus VP6 proteins from CV-1 cells by flow cytometry (Figure 1B–H). As expected, compared to mock infected cells, CV-1 cells infected with the WT virus showed a VP6 signal (Figure 1B,C). We failed to generate recombinant rotaviruses that contained modified NSP2 or NSP4, as VP6-positive cells were not observed after infecting CV-1 cells with the corresponding reverse genetics reactions (Figure 1E,G). In contrast, double-positive cell populations that expressed VP6 and GFP were observed when the rescue reactions contained modified NSP1, NSP3, or NSP5, indicating that we successfully generated a recombinant rotavirus with a GFP insertion at each of these locations (Figure 1D,F,H). We thus found three positions for heterologous gene insertion in rotavirus genome. Among NSP1, NSP3, and NSP5, modified NSP1 and NSP5 led to a higher percentage of VP6/GFP double positives (Figure 1D), suggesting a higher efficiency of virus rescue. 

**Figure 2:**
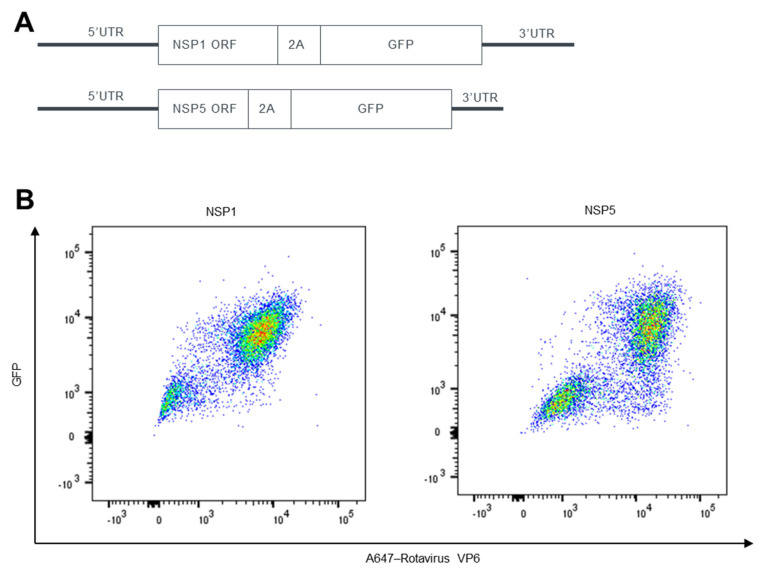
Generation of rSA11 strains expressing GFP at the C terminus of NSP1 or NSP5 without 3’ ORF repeats. (**A**) Schematic representation of plasmids used for the recovery of rSA11 viruses encoding GFP downstream of NSP1 or NSP5. (**B**) Representative flow cytometry analysis of CV-1 cells infected with rSA11 strains generated with plasmids shown in (**A**). Expression levels of GFP and rotavirus protein VP6 were examined. Pseudocolor plots were shown using color to denote areas of high and low population density.

We then deleted the NSP1 and NSP5 ORF 3′ sequence repeat after the stop codon to avoid potential recombination events (Figure 2A). We still observed the double-positive cell population that expressed GFP and VP6 (Figure 2B), indicating virus packaging does not require the additional repeated sequence. Similar to recombinant rotaviruses rescued in Figure 1, after deleting the repeated sequence, the NSP1 position still resulted in a higher percentage of VP6/GFP double-positive population than NSP5 (Figure 2B). We thus focused on viruses containing GFP downstream of NSP1 without a 3′ sequence repeat for further characterization and referred to it as rSA11-GFP. 

Using flow cytometry to examine VP6 expression, we also established a flow-cytometry-based infectivity assay for determining rotavirus titers (Supplementary Figure S1). After infection, CV-1 cells were cultured in media supplemented with FBS, but not trypsin, to prevent multiple rounds of infection. Based on Poisson distribution, the multiple of infection (MOI) can be calculated after determining the percentage of VP6-positive cells. The titer, as infectivity unit per ml (IU/mL), can then be calculated based on the number of cells and the amount of viral stock used at infection. 

### 3.2. Characteristics of rSA11-GFP

**Figure 3:**
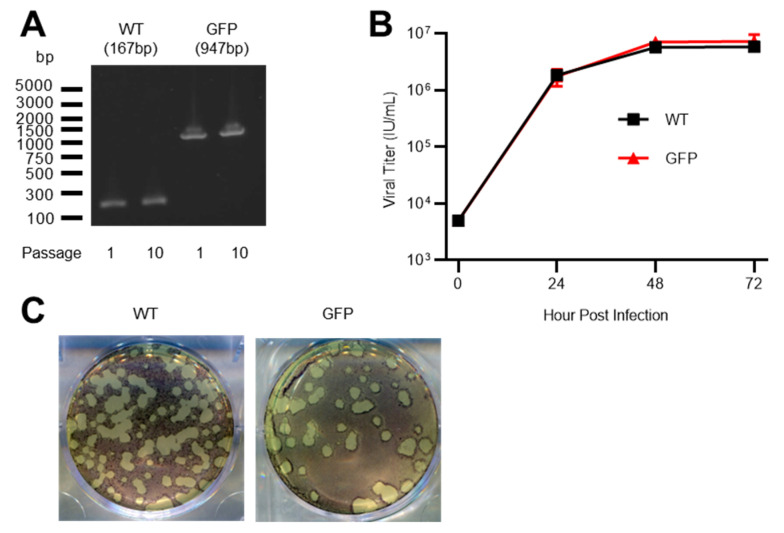
Properties of rSA11-GFP. (**A**) Genetic stability of rSA11-GFP. rSA11 and rSA11-GFP were serially passaged ten times on MA104 cells and analyzed by RT-PCR using primers flanking the insertion site. The expected band sizes are indicated in parentheses. (**B**) Growth kinetics of rSA11 and rSA11-GFP. MA104 cells were infected with viruses at an MOI of 0.01 IU/cell and harvested at indicated time points. Virus titer was determined in a flow cytometry-based infectivity assay using CV1 cells. Data are expressed as the mean and range of duplicates. (**C**) Plaque formation on MA104 cells by rSA11and rSA11-GFP. Data representative of three independent experiments.

To examine the genetic stability of rSA11-GFP, we passaged rSA11-GFP and rSA11-WT on MA104 cells ten times, extracted viral RNA from passage one (reverse genetics product) and ten, and performed RT-PCR using primers flanking the insertion site. RT-PCR products were visualized by gel electrophoresis and sequenced by Sanger sequencing. Fragments migrated to expected sizes (Figure 3A), and sequencing reactions showed that no mutations were generated for ten passages. Our results thus indicated that rSA11-GFP was genetically stable.

We also compared the growth kinetics of rSA11-GFP with rSA11-WT. MA104 cells were infected with viruses at an MOI of 0.01 IU/cell and harvested at 24, 48, and 72 h post infection (Figure 3B). The growth curves of rSA11-GFP and rSA11-WT were indistinguishable, indicating that the insertion of GFP did not affect the fitness of the recombinant virus in vitro. In addition, plaques formed by rSA11-GFP and rSA11-WT were of similar sizes (Figure 3C), further supporting that the insertion of GFP downstream of NSP1 had no effects on rotavirus replication. 

### 3.3. rSA11-GFP Based Microneutralization Assay

**Figure 4:**
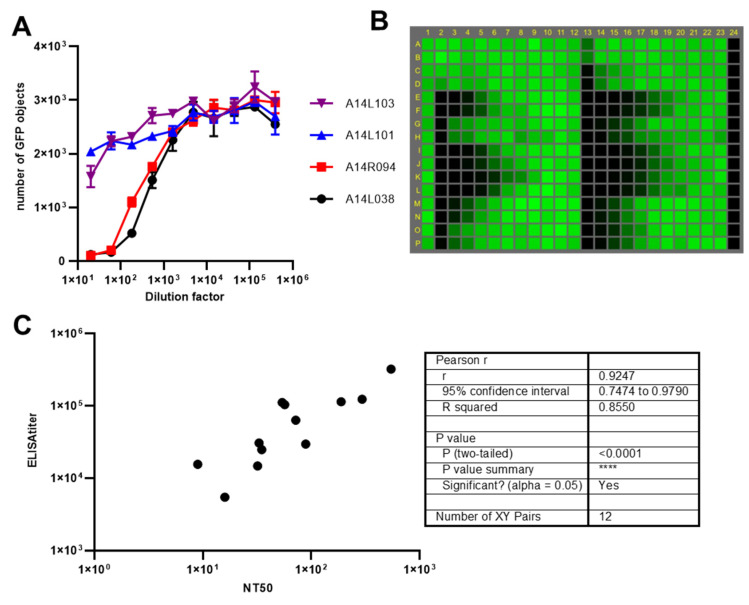
rSA11-GFP based microneutralization assay. (**A**) Representative serum neutralization curves of four rhesus monkeys. rSA11-GFP, preincubated with serial diluted serum samples, was used to infect CV-1 cells. After overnight incubation, numbers of GFP positive cells were numerated. Median is shown from triplicated wells. (**B**) The conversion of the neutralization assay to a high-throughput platform. rSA11-GFP, preincubated with serial diluted serum samples, was used to infect CV-1 cells. After overnight incubation GFP positive cells were numerated by Acumen. A representative review of a 384-well plate is shown. The plate map is provided in Supplemental Table S1. No serum samples were used in the first column and the last column contained mocked infected cells. Serum samples were diluted threefold with 11-point titration. Wells are colored based on the numbers of GFP positive cells. Average for no serum, infected controls was ~100 objects/well while average for cells only was ~3. (**C**) The correlation of neutralization titers and ELISA titers of serum samples from 12 African green monkeys and its statistical analysis. **** means *p* value less than 0.0001.

Because traditional neutralization assays, such as plaque reduction neutralization test (PRNT) and fluorescent foci reduction neutralization test (FRNT), that rely on antibody staining are time-consuming and labor intensive, we sought to develop a microneutralization assay based on the GFP signal using rSA11-GFP. It is known that immunity against rotavirus exists naturally in some monkey colonies [20]. We examined four rhesus monkey serum samples in the 96-well format microneutralization assay and found that, as expected, all four samples neutralized rSA11-GFP, with two showing higher neutralizing capacity (Figure 4A). 

We then converted the assay to a higher throughput format by adapting it to 384-well plates and eliminating the CV-1 pre-seeding step (Figure 4B and Supplementary Table S1). In the 384-well plate format, CV-1 cells in suspension were applied directly to the virus/serum mixtures for infection. We used this high-throughput assay to examine 12 African green monkeys in our animal facility (Figure 4C). All animals showed pre-existing antibodies against rotavirus based on neutralization assay and IgG ELISA assay that used SA11 for coating. We observed a wide range of antibody titers with NT_50_ titers ranging from 9 to 545 and ELISA titers ranging from 5444 to 323,096. Our results indicated that all African green monkeys we examined were seropositive for SA11. It is unlikely that we are detecting maternal antibodies as all monkeys are 2–3 years old. To verify that the neutralization ability was antibody-dependent, we performed polyclonal IgG purification on the serum samples from seven African green monkeys using protein G beads. We obtained similar titers before and after purification while the flow-through showed limited neutralization capacity (Supplementary Figure S2). The high level of correlation (r = 0.9247, *p* < 0.0001) between the neutralization titer and ELISA titer (Figure 4C) suggested that either almost all antibodies captured by ELISA were neutralizing antibodies or the proportions of rotavirus antibodies with neutralization capacity were consistent among African green monkeys. 

### 3.4. Pre-Existing Immunity in Human and Other Animal Species

**Figure 5:**
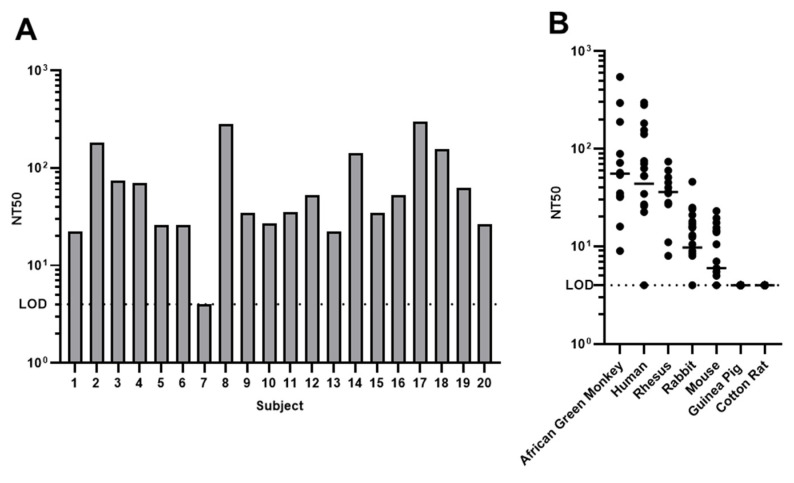
Pre-existing immunity in human and other animal species. (**A**) Serum neutralization titers of 20 human donors. (**B**) Serum neutralization titers of animal samples from indicated species. The bars indicate the median.

We next determined neutralizing antibodies in human donors by the rSA11-GFP-based microneutralization assay (Figure 5A). Group A rotavirus contains more than 40 G (VP7) serotypes and more than 55 P (VP4) serotypes according to Rotavirus Classification Working Group (RCWG [21]). SA11 was originally isolated from a healthy African green monkey and belongs to G3P5B[2]. Serotypes G1, 2, 3, 4, 9, and 12 are epidemiologically important for human. Although both VP4 and VP7 can elicit neutralizing antibodies, this assay specifically reveals G3-specific antibodies as there is no known P5B[2] human strain. Out of the twenty samples we initially examined, only one did not show a neutralization titer above the limit of detection, suggesting the wide prevalence of G3 antibodies in human population. The titers were similar to those of African green monkeys in the animal facility and higher than those of the 11 rhesus monkeys we examined. We further tested 30 human serum samples by ELISA binding and neutralization assays (Supplementary Figure S3). As expected, we observed a weaker correlation between the neutralization titers and ELISA titers from human (r = 0.5322, *p* = 0.0025) compared to African green monkey. SA11 is a simian RV origin isolated from an asymptomatic African green monkey. African green monkeys are potentially infected with strains similar to SA11 naturally, while there are many human rotavirus strains with different serotypes. The weaker correlation revealed the complexity of human rotavirus immune status as repeated infections are common, with secondar infections often involving different serotypes [22]. The observation that all rhesus monkeys examined showed neutralization titers could be a result of natural infection as rhesus monkey rotavirus RRV also belongs to G3 [23]. It is worth noting that the possibility of heterotypic neutralization cannot be ruled out, as multiple studies of human and murine neutralizing antibodies showed heterotypic immunity against both VP7 and VP4 [24,25,26,27].

Rotaviruses are a group of viruses impacting a variety of animals, including common animal models used for vaccine or drug development. The microneutralization assay also allowed us to evaluate rabbit, mouse, guinea pig and cotton rat serum samples (Figure 5B). Several rabbit rotaviruses are G3 serotype viruses, and we indeed revealed neutralizing antibodies in many of the rabbits (15 out of 22), although the titers were much lower than those of human or simian origin. Although there is no known mouse rotavirus strain in the same serotype as SA11 based on VP4 and VP7, we observed neutralization titers in some of the mouse serum samples (16 out of 25) that could be caused by heterotypic immunity or undiscovered mouse strains. Guinea pig and cotton rat are widely used in infectious disease and vaccine research. No rotavirus has been reported in those two species. We did not discover any neutralizing antibodies against SA11 in any guinea pig or cotton rat we examined. In summary, the rSA11-GFP-based microneutralization assay enabled us to evaluate pre-existing immunity in several animal species, including humans, in a high-throughput manner.

## 4. Discussion

We extend prior studies [5,7] on rotavirus reverse genetics systems to demonstrate three positions in monkey rotavirus strain SA11 genome that are permissive for the insertion and expression of heterologous genes. Using SA11 expressing GFP, we develop a high-throughput microneutralization assay that enables a rapid evaluation of pre-existing antibodies in different animal species, including humans.

Our results show that rotavirus nonstructural proteins NSP1, NSP3, and NSP5 can tolerate heterologous gene insertion at their C-termini without altering any rotavirus protein-coding sequences. The original publication on rotavirus reverse genetics explored the modification of the C-terminus of NSP1 to express reporter proteins by partially deleting NSP1 protein to accommodate split GFP fragment GFP11 or NanoLuc [5]. In our strategy, we encoded the entire GFP after NSP1 along with a 2A cleavage sequence while keeping NSP1 intact as it plays a role in intestinal viral replication, pathogenesis, and transmission [28]. In another publication on reverse genetics for rotavirus, the C-terminus of NSP3 was fused to heterologous antigens, including partial SARS-CoV-2 spike protein [14,15]. However, our experiments showed that rescuing NSP3-GFP was less efficient compared to NSP1-GFP and NSP5-GFP, suggesting that the insertion of foreign sequences at this position renders rotavirus more genetically unstable. The largest insertion at NSP3 has been around 2 kb [15]. NSP1 and NSP5 positions may tolerate larger insertions, increasing the capability of rotaviruses as an expression platform. Additionally, for the first time, we demonstrate that NSP5 can also be manipulated to express a heterologous gene.

Neutralizing antibodies play a crucial role in the humoral immune response. Traditional rotavirus neutralization assays, such as the plaque reduction neutralization test (PRNT) or fluorescent foci reduction neutralization test (FRNT), are laborious and time-consuming, limiting the in-depth examination of neutralization antibodies in human and animal models. Microneutralization assays based on GFP or other reporter proteins have been established for a variety of viruses, including human metapneumovirus, human cytomegalovirus, and respiratory syncytial virus [29,30,31]. These assays support a high-throughput format compatible with robotic system, facilitating basic research and vaccine development.

Neutralizing antibodies against rotaviruses have been discovered against VP4 and VP7, two viral proteins that determine the serotype. Certain antibodies specific to VP6 have demonstrated the ability to neutralize rotaviruses intracellularly [32]. In our study, we developed a microneutralization assay based on rSA11-GFP, a serotype G3P5B[2] virus. The same concept and method can be applied to interrogate other serotypes. Strains, including several human strains (KU [8], Odelia [7], CDC-9 [6] and HN126 [9]), have been rescued by reverse genetics, and chimeric rotaviruses bearing VP4 and VP7 from clinical isolates have been generated using SA11 as the backbone [12]. Alongside our findings, these results imply the possibility of either constructing rotaviruses from different serotypes expressing GFP or generating a panel of rotaviruses containing SA11 NSP1-GFP but with VP4, VP7 and VP6 from other strains/serotypes to tease out neutralizing antibodies against each component in animal and human samples. Viruses with different serotypes expressing other reporter proteins, such as BFP and RFP, can potentially be used in parallel with a GFP virus, enabling multiplexed neutralization assays that detect NT50s against more than one serotype in one assay.

The high level of correlation between ELISA titers and neutralization titers in our African green monkey experiments suggest that the microneutralization assay can be used as a surrogate assay for ELISA. By using this assay, we can bypass the requirement of species-specific secondary antibodies in ELISA and compare neutralizing titers of sera from different animal species. Consistent with previous reports [20], all simians we tested showed neutralization titers above the limit of detection. Interestingly, the neutralizing titers of human samples are similar to those of simian with only nine negative samples out of 50, indicating the prevalence of G3-serotype-specific antibody in humans. In contrast, no neutralizing antibodies were detected in cotton rats and guinea pigs, while more than half of the mouse samples contained neutralizing antibodies above the limit of detection, although the titers are lower than those of rabbit, in which several rotaviruses are of G3 type. Our results suggest either heterotypic neutralizing immunity is prevalent in mice, or there might be mouse rotavirus strains with serotypes G3 or P5B[2] that have yet to be discovered.

A high-throughput method to measure neutralizing antibodies enables two types of studies. First, once a panel of recombinant rotaviruses with different serotypes containing NSP1-GFP is established, it provides a toolset for seroepidemiology studies to characterize the prevalence of different serotypes of rotavirus in humans. Second, the method also facilitates the selection of appropriate animal models for preclinical studies. These studies will provide insights into the mechanisms underlying the low efficacy of rotavirus vaccines in developing country, enabling the rational design of next-generation rotavirus vaccines. Importantly, although SA11 is unlikely to be a suitable platform, now that it is established that rotavirus can express heterologous proteins, these studies will explore the potential of using rotaviruses as a vector for delivering vaccine antigens or therapeutic payloads.

## Data Availability

The data presented within this study are available within the manuscript.

## Supplementary Materials

The following supporting information can be downloaded at: https://www.mdpi.com/article/10.3390/v15102034/s1, Figure S1: Flow cytometry-based infectivity assay; Figure S2: Neutralization titers of African green monkey serum samples before and after antibody purification; Figure S3: The correlation of neutralization titers and ELISA titers of 30 human serum samples and its statistical analysis; Table S1: A sample 384-plate map.
