# Supplementary material for: A Novel Rotavirus Reverse Genetics Platform Supports Flexible Insertion of Exogenous Genes and Enables Rapid Development of a High-Throughput Neutralization Assay
Source: Viruses. 2023 Sep 30;15(10):2034. doi: 10.3390/v15102034 (PMC10611407; doi:10.3390/v15102034)
Supplement: Supplementary file 1 [file viruses-15-02034-s001.zip › 29Sep2023rotapaper_preexisting_sup.pdf]

Sup Figure S1

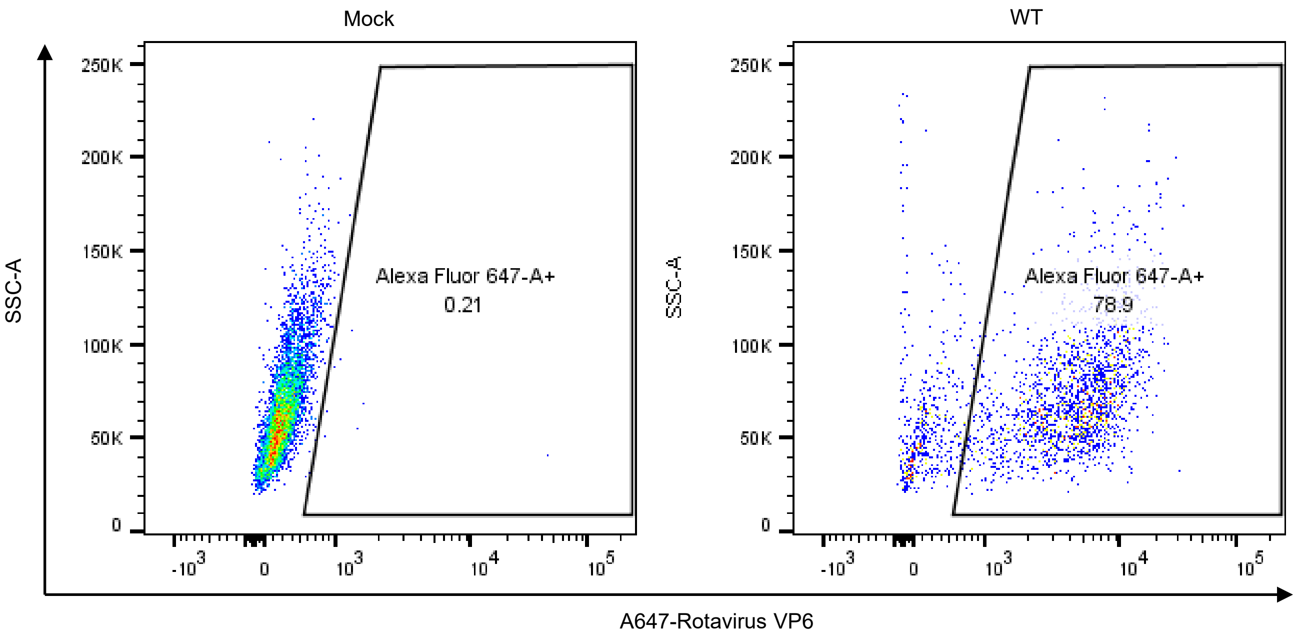

Supplementary Figure S1. Flow cytometry-based infectivity assay. Mock infected cells (left) and rSA11 infected cells (right) were stained with rotavirus VP6 antibody. MOI was calculated using the percentage of VP6 positive cell population base on Poisson distribution. IU/ml was calculated by IU/ml = (# of cells at infection) × [MOI / (ml of viral stock used at infection)]

Confidential

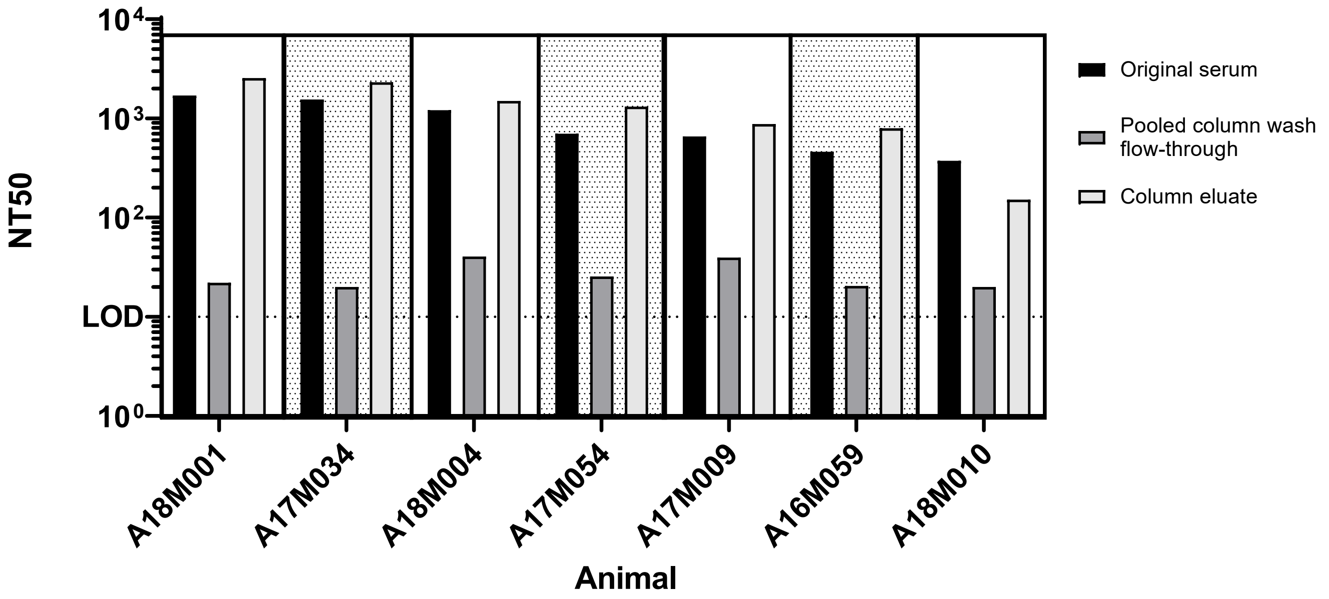

Supplementary Figure S2. Neutralization titers of African green monkey serum samples before and after antibody purification. Seven African green monkey serum samples were purified with protein G. Original samples, wash flow-through and column elutes were examined by the GFP based neutralization assay.

C Confidential

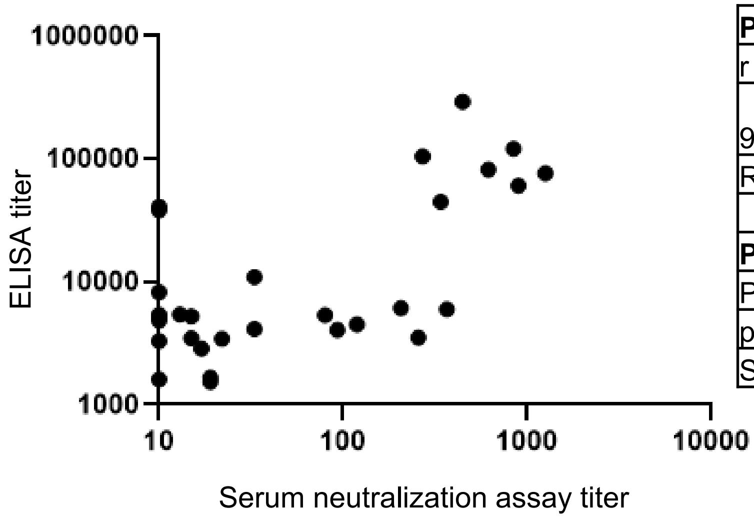

Pearson r	
r	0.5322
95% confidence interval	0.2127 to 0.7489
R squared	0.2833
**P value**	
P (two-tailed)	0.0025
p value summary	**
Significant?	Yes

Supplementary Figure S3. The correlation of neutralization titers and ELISA titers of 30 human serum samples and its statistical analysis.

Confidential
